# Detachable dissolvable microneedles: intra-epidermal and intradermal diffusion, effect on skin surface, and application in hyperpigmentation treatment

**DOI:** 10.1038/s41598-021-03503-5

**Published:** 2021-12-16

**Authors:** Pritsana Sawutdeechaikul, Silada Kanokrungsee, Thanyapat Sahaspot, Kamonwan Thadvibun, Wijit Banlunara, Benchaphorn Limcharoen, Titiporn Sansureerungsikul, Teeranut Rutwaree, Miranda Oungeun, Supason Wanichwecharungruang

**Affiliations:** 1 Department of Chemistry, Faculty of Science, Chulalongkorn University, Bangkok, Thailand; 2 Faculty of Medicine, Skin Center, Srinakharinwirot University, Bangkok, Thailand; 3 Department of Pathology, Faculty of Veterinary Science, Chulalongkorn University, Bangkok, Thailand; 4 Department of Anatomy, Faculty of Veterinary Science, Chulalongkorn University, Bangkok, Thailand; 5 Mineed Technology, 142 Innovation Cluster 2, Thailand Science Park, Pathum Thani, Thailand; 6 Department of Petrochemistry and Polymer Science, Faculty of Science, Chulalongkorn University, Bangkok, Thailand; 7 Center of Excellence in Materials and Bio-Interfaces, Chulalongkorn University, Bangkok, Thailand

**Keywords:** Medical research, Materials science

## Abstract

Delivering bioactive compounds into skin tissue has long been a challenge. Using ex vivo porcine and rat skins, here we demonstrate that a detachable dissolvable microneedle (DDMN) array, a special dissolvable microneedle that allows needle detachment from the base within 2 min post administration, can effectively embed a model compound into epidermis and dermis. Diffusion of the compound from the needle embedding sites to the nearby skin tissue is demonstrated at various post administration periods. The relationship between the time that a conventional dissolvable microneedle array is left on skin without needle detachment from the base and the degree of skin surface abrasion at each microneedle penetration spot is also demonstrated on skin of human volunteers. Co-loading glutathione with vitamin C (vitC) can stabilize vitC in the DDMN. DDMN loaded with vitC and glutathione can help erasing post-acne-hyperpigmentation spots.

## Introduction

Unlike plant and most other animals, human and a few other primates including the Indian fruit-eating bat and the guinea pig, lack l-gulono-gamma-lactone oxidase, therefore, cannot convert glucose into ascorbic acid or vitamin C (vitC). As a result, ingestion of vitC is health essential for these species.

VitC is a potent antioxidant that has been used topically in dermatology to treat and prevent changes associated with photo-aging such as wrinkle and hyperpigmentation^1,2^. VitC regulates cellular collagen level by acting as an essential cofactor for the actions of prolyl hydroxylase and lysyl hydroxylase in the collagen crosslinking process^3^. In addition, vitC directly regulates DNA involved in collagen synthesis, stabilizes procollagen messenger RNA^4^, and increases the synthesis of several specific lipids of skin surfaces^5^. An in vitro experiment showed that in an absence of vitC, fibroblast cells obtained from elderly volunteers proliferated at 1/5 the rate of those obtained from newborns. However, in the presence of vitC, the elderly cells proliferated better than the newborn fibroblasts. The newborn fibroblasts also proliferated four times better when exposed to vitC. Both newborn and elderly cells synthesized more collagen in the presence of vitC^6^. This vitamin interacts with copper ions at an active site of tyrosinase enzyme, preventing the transformation of tyrosine into melanin pigment by the enzyme, resulting in skin whitening effect^7,8^. Unfortunately, oral administration of vitC is not effective enough to achieve skin hypopigmentation. Limited vitC absorption in the intestine and un-targetable distribution of vitC, are the two main reasons^7,9^. An ideal strategy to lessen skin hyperpigmentation is to directly supply skin tissue with vitC. To do so, vitC instability in topical formulations, and limited skin penetration of the compound, must be overcome.

VitC is easily degraded by various factors such as light, oxygen, high temperature, alkali, and a presence of some heavy metals^7^. Reported strategies to stabilize vitC include encapsulation^10^, pH adjustment^11^, conjugation with squalene^12^, and inclusion of electrolytes and other antioxidants into formulations^13^. Nevertheless, vitC stabilization in topical formulations is still a challenge. More stable derivatives of vitC such as ascorbyl‐6‐palmitate, 3-O-ethyl-ascorbic acid^14^, magnesium ascorbyl phosphate^15^, ascorbyl tetraiso‐palmitate^16^, ascorbyl glucoside^17^, and trisodium ascorbyl 6-palmitate 2-phosphate^18,19^, have been synthesized and used in cosmetic formulations. Unfortunately, limited chemical stability, limited skin permeation and inefficient conversion into the active vitC in skin have been reported for these derivatives^19–21^. VitC itself demonstrates poor skin absorption when used in normal cosmetic formulations with skin compatible pH of 5–6^20,22^. At pH of less than 4, vitC molecule is neutral because all hydroxyl groups on vitC molecule are not deprotonated, and the molecule can be more easily absorbed into the skin^20,22^. This, however, contradicts the recommended non-irritating pH range for skincare products. Controlled laser ablation of stratum corneum can improve vitC penetration into skin^23^, but the treatment requires laser machine and an expert. Therefore, there is an evident interest to develop an efficient and convenient strategy to send vitC across stratum corneum into skin tissue.

The concept of microneedles (MNs) was patented in 1976^24^, and such array of micro-dimensioned needles was fabricated from silicon two decades later by high precision microelectronic tooling^25^. The device, today known as solid microneedles (solid MNs), has been used to generate skin channels prior to an application of drug solution. The later developed concept of dissolving MNs or DMNs is that the needles are made of biocompatible/water-soluble materials so that the needles can fully dissolve in skin tissue, using interstitial fluid as solvent^26^. Drug can be loaded into the needles, therefore, the DMNs can be used to deliver drug into skin tissue. Popular materials used as needle structure include hyaluronic acid (HA), gelatin, sodium alginate, poly-γ-glutamic acid, polyvinylpyrrolidone (PVP) and polyvinyl alcohol (PVA)^27^.

Despite the sound good concept of DMNs, administration of the device requires time for the dissolution of the skin-embedding needles before removing the base of the needle array. Incomplete dissolution of the embedded needles leads to a removal of undissolved part, and results in less drug being delivered than expected. In addition to these problems, production of DMNs with precise drug at only the needle part still face a few challenges including the need of centrifugation step which add cost to manufacturing, and the high drug lost during manufacturing which makes it impractical for expensive drugs. As a result, DMN has not been well accepted in medical application regardless of a recent report on a long-term safety of repeating uses of DMNs in human volunteers^28^.

Strategies to enable complete delivery of drug loaded DMNs into skin tissue without having to leave a DMN base on the skin for a long time, have been reported recently. Examples include putting into the needles magnesium nanoparticles that will react explosively with interstitial fluid^29^, creating of the DMNs with fast dissolving drug-loaded needle tips^30^, fabricating of needles that can swell/dissolve upon the application of electricity^31^, making needles to be heat responsive^32^, and using acid–base reaction that can generate gas at the junction between needles and the base^33^.

Our lab has also reported the design and fabrication of DMNs with water penetrable backing or the so called detachable dissolvable microneedles (DDMNs) that allow quick (1–2 min) detachment of needles from the base^34–36^.

Although it has been assumed that the drug loaded DMNs can dissolve and diffuse in skin tissue, the diffusion has never been directly demonstrated. Here via ex vivo experiments using porcine and rat skins, we show the dissolution and diffusion of dye molecules (used as a model drug) in epidermis and dermis at various times after their administration into the tissue via the embedment of the dye-loaded DDMNs. The relationship between the period that the microneedle array is placed on skin and the skin surface damage, was also investigated on human volunteers, and is reported here. We also show the optimization of DDMN formulation to stabilize vitC, and the use of the optimized formulation to treat facial post-acne-hyperpigmentation spots in human volunteers. Effect of microneedle length on the efficacy is also reported.

## Results

### Vitamin C stability, mechanical property and skin penetration ability of DDMNs

**Figure 1:**
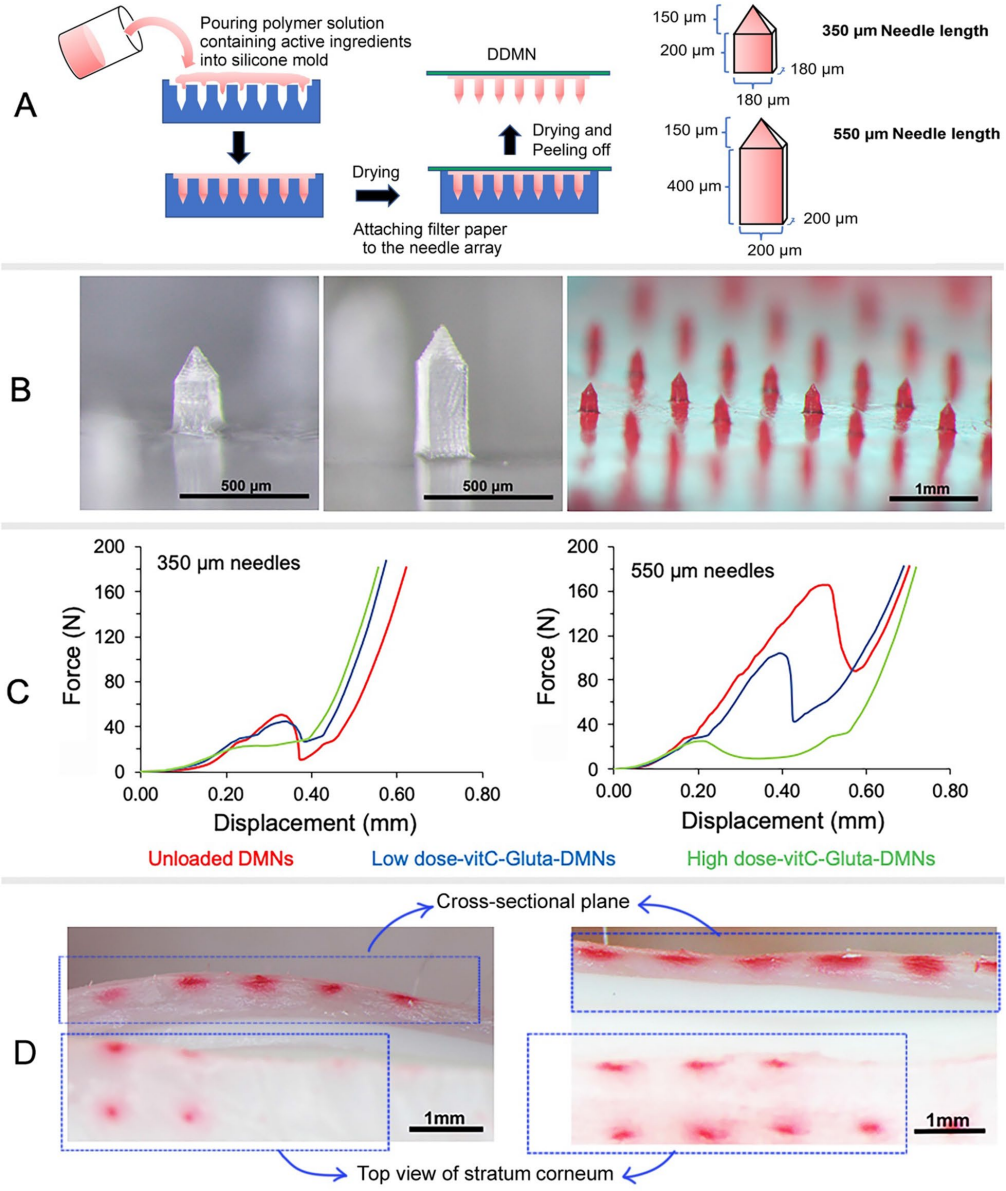
Fabrication process, morphology, mechanical property and skin penetration ability of DDMNs: (**A**) DDMN fabrication process and needle dimensions; (**B**) Representative stereomicroscopic images of the low dose-vitC-gluta-DDMN of 350 µm (left) and 550 µm (middle) needle length and red dye-spiked low dose-vitC-gluta-DDMNs of 350 µm needle length (right); (**C**) Graphs of displaced distances and applied forces for various DMNs with the needle length of 350 µm (left) and 550 µm (right); (**D**) Representative stereomicroscopic images of the surgically cut porcine ear skin immediately after the administration of red dye-spiked low dose-vitC-gluta-DDMNs with the needle length of 350 µm (left) and 550 µm (right).

Experiments to find an additive to stabilize vitC in the DDMNs resulted in the use of glutathione at 25% of the amount of vitC (see detailed result and discussion on the additive finding in Supplementary Section, Supplementary Fig. S1). We fabricated 350 and 550 µm DDMNs with the square base nail-shaped needles with two loading percentages of vitC, the 20% vitC + 5% glutathione (low dose-vitC-gluta-DDMN patch) and the 40% vitC + 10% glutathione (high dose-vitC-gluta-DDMN patch), via the micro-molding technique using the mixture of hyaluronic acid (HA), polyvinyl alcohol (PVA) and sucrose at 0.5:1:1 weight ratio as needle base material (Fig. 1A,B left and middle). Control DDMN patches (unloaded DDMNs), were also prepared. Graphs between compressive force and displaced distance of the prepared DMN patches are shown in Fig. 1C. The ex vivo skin penetration experiment was conducted using the low dose-vitC-gluta-DDMN patch of 350 and 550 µm needle lengths that contained red dye (Fig. 1B right) and the results are shown in Fig. 1D.

### Dissolution and diffusion of microneedles in ex vivo skin tissue

**Figure 2:**
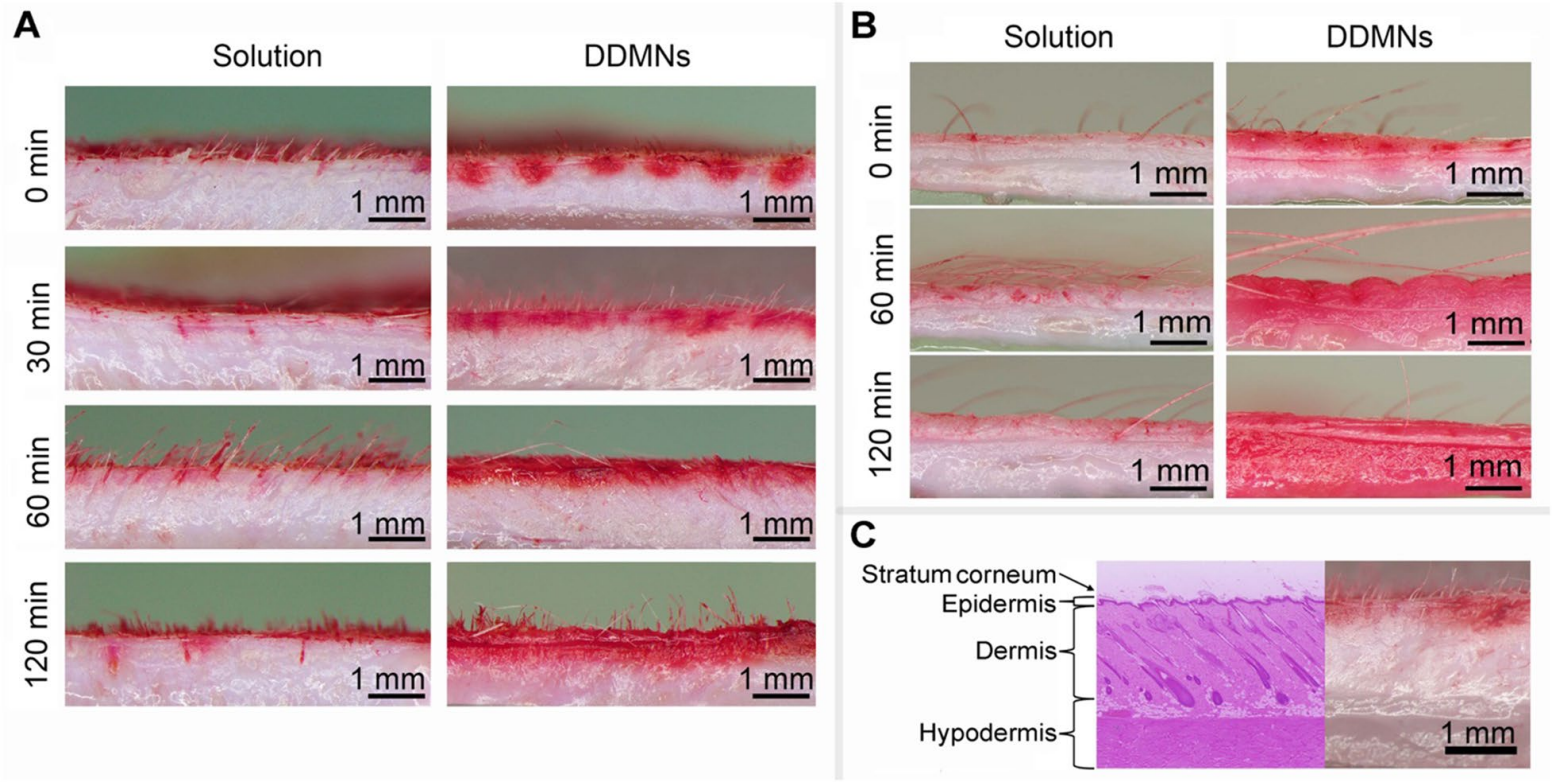
Stereomicroscopic images of cross sectioned skin tissues at defined times (denoted at the left side of each row) after the topical administration of red dye solution at 470 µg dye on circular area of 1.5 cm diameter of the skin (left cloumn of (**A,B**)) and red dye-loaded DDMNs (containing 470 µg dye in circular patch of 1.5 cm diameter, 550 µm needle length) (right column of (**A,B**)) for the rat back skin (**A**) and pig ear skin (**B**). Figure (**C**) shows layers of rat skin.

Results of the ex vivo intra-epidermal and intradermal deliveries of 0.47 mg of red dye by the 550 µm circular DDMN patch of 1.5 cm diameter are shown in Fig. 2A for the rat back skin, and in Fig. 2B for the pig ear skin. Results of topical application of the solution containing the same amount of red dye to the circular area of 1.5 cm diameter of the ex vivo rat back and pig ear skins are also shown. The results are presented as images of the cross sectioned skin tissues at various post application times. Figure 2C shows layers of rat skin. As shown in Fig. 2A for the rat skin, at 0 min (immediately after DDMN application), red color appeared in an array of spots with gap between spots matching gap between needles on the DDMNs and each red spot spans from stratum corneum to the dept of approximately 0.4–0.5 mm (epidermis and upper dermis). The image of tissue at 30 min indicates red dye diffusion in the horizon direction (closing the gap previously observed at 0 min). Red dye in rat skin spans the dept of 0.4–0.5 mm for the 30 and the 60 min time points. The red zone expands to the dept of 0.6 mm at 120 min (Fig. 2A). In the case of pig skin (Fig. 2B), red dye covers the dept of approximately 0.75 and 1 mm at 60 and 120 min, respectively (Fig. 2B).

### In vivo skin irritation in rats

**Figure 3:**
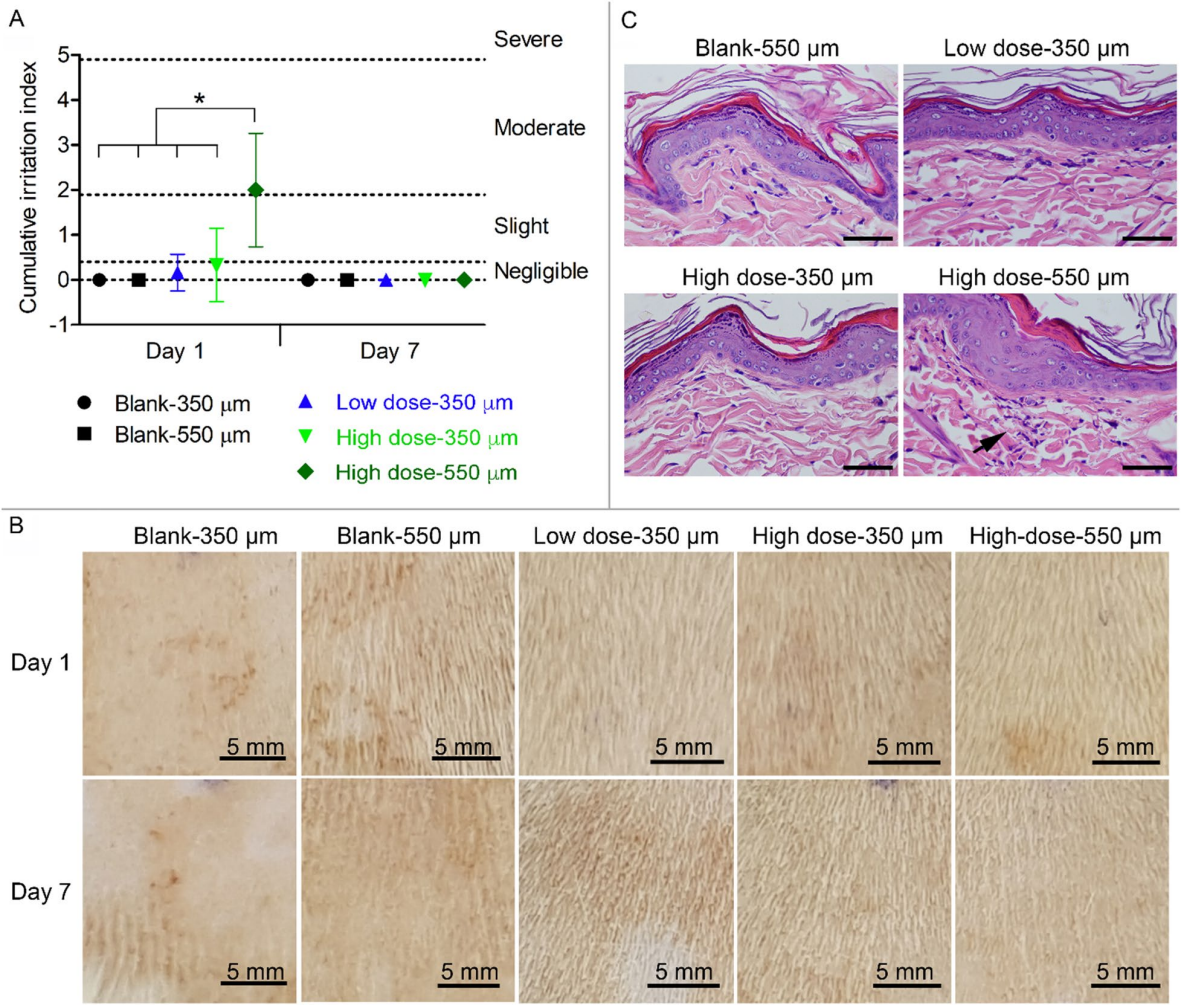
In vivo skin irritation. (**A**) Cumulative irritation indexes from the rat skin at day 1 and day 7 after single DDMN administration. Statistical analysis was carried out using one-way ANOVA followed by Tukey post hoc test at the significant level of p < 0.05, and *represents significant difference. Data are shown as mean with the error bars represent standard deviation obtained with n = 3 for unloaded DDMNs (Blank-350 µm and Blank-550 µm); and n = 6 for low dose-vitC-gluta-350-DDMNs (Low dose-350 µm), high dose-vitC-gluta-350-DDMNs (High dose-350 µm) and high dose-vitC-gluta-550-DDMNs (high dose-550 µm). (**B**) Macroscopic images of the rat back skin at the treated sites at day 1 (Top row) and day 7 (Bottom row) after single DDMN administration. (**C**) Representative histopathological images of the cross-sectioned skin tissues at the treated site of the rats, collected 1 day after the single DDMN administration. Black arrow points at the infiltrated mononuclear cells. Scale bar represents 50 µm.

Skin irritation at the back of rats was tested for (1) low dose-vitC-gluta-350-DDMN patch, (2) high dose-vitC-gluta-350-DDMN patch, (3) high dose-vitC-gluta-550-DDMN patch, and (4 and 5) the unloaded DDMNs of 350 and 550 μm needle lengths (blank-350 µm and blank-550 µm). Skin irritation indexes and representative photographs of skin at the administered sites (at day 1 and day 7 post the single administration), are shown in Fig. 3A,B respectively. Histopathological evaluation of cross-sectional views of epidermis and dermis at day 1 and day 7 post single administration revealed no skin irritation for blank-550-DDMN patch, low dose-vitC-gluta-350-DDMN patch, and high dose-vitC-gluta-350-DDMN patch, i.e., skin tissue appeared normal, no abnormal inflammatory cell infiltration was observed (Fig. 3C, Supplementary Fig. S2). Only on day 1 post application, small numbers of mononuclear cells were found in skin tissue treated with high dose-vitC-gluta-550-DDMNs (black arrow in Fig. 3C), but not on day 7 (Supplementary Fig. S2D).

### Skin surface after administration

**Figure 4:**
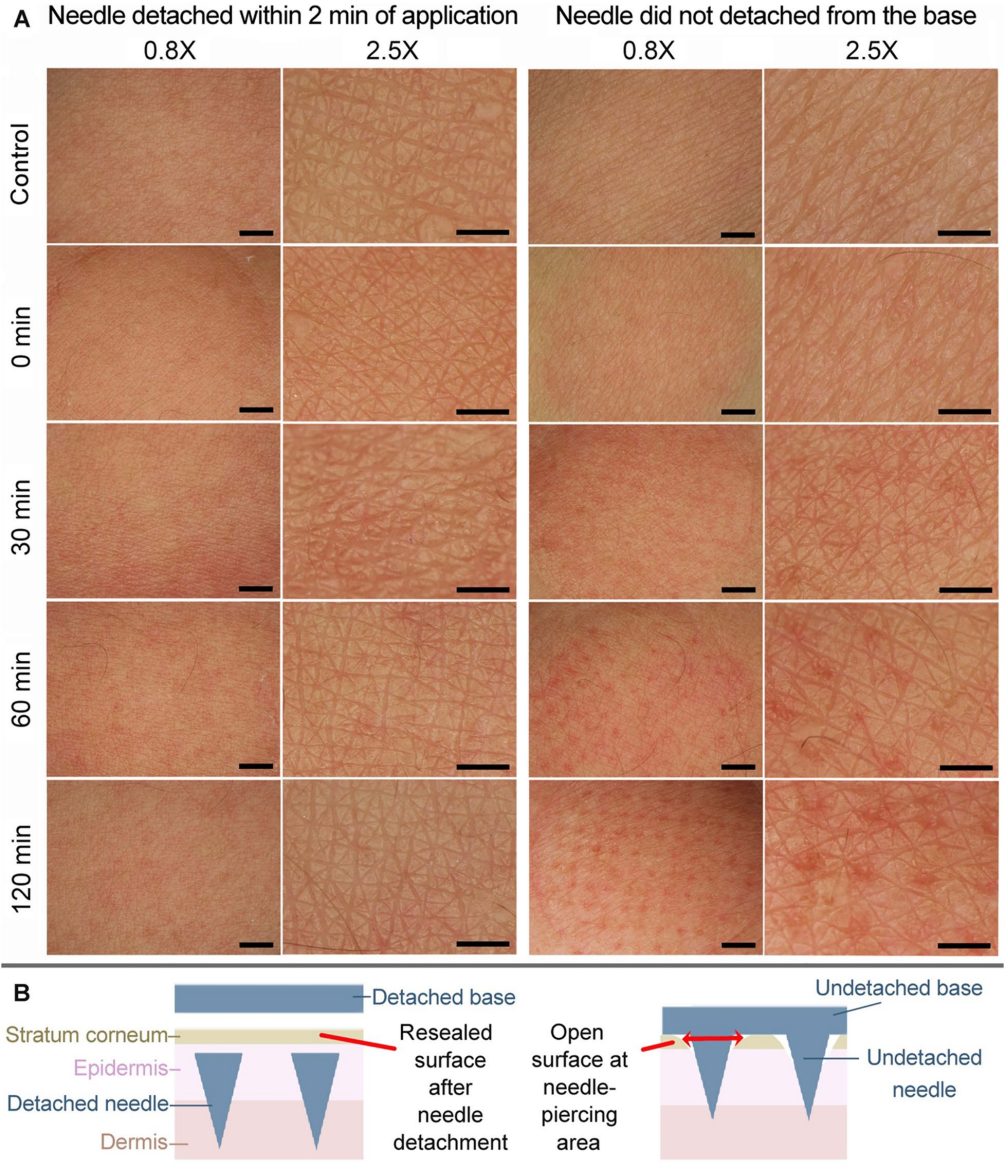
Skin surface conditions after microneedle administration. (**A**) Photographs at 0.8× (first and third columns from the left) and 2.5× (second and fourth columns from the left) magnifications of a human volunteer inner forearm skin after the application of the two types of 350 μm microneedles, the needle-detaching type and the non-detaching type. *For the DDMN or the needle-detaching type*, the skin was applied with the low dose-vitC-gluta-350-DDMNs (the needles were detached, and the base removed, at 2 min after the administration), then the skin was photographed at various times post the administration (see the denoted times on the left of each row). *For conventional microneedles or the DMN with no needle detaching feature,* the skin was applied with the 350 μm DMN patch (same morphology as the DDMNs) and the patch was left on the skin for 0, 30, 60 and 120 min (time denoted on the left of each row), then the patch was removed, and the skin was photographed immediately. Control is the image of the untreated skin. Scale bars on images in the first and the third columns from the left represent 2 mm, and in the second and the fourth columns represent 1 mm. (**B**) Illustration showing how needle detachment allows for skin surface resealing (Left), and how the un-detachment of needles creates skin channels at each needle penetration site (Right).

Images of the skin surface after the low dose-vitC-gluta-DDMN application over the inner arm area of human volunteers are shown in the left side of Fig. 4A and Supplementary Fig. S3 for the 350 and 550 µm needle lengths, respectively. Skin surface after the application of conventional microneedles (no needle-detaching feature, same needle morphology as the DDMNs) are shown in the right side of Fig. 4A and Supplementary Fig. S3 for the 350 and 550 µm needle lengths, respectively.

### Post-acne-hyperpigmentation

**Figure 5:**
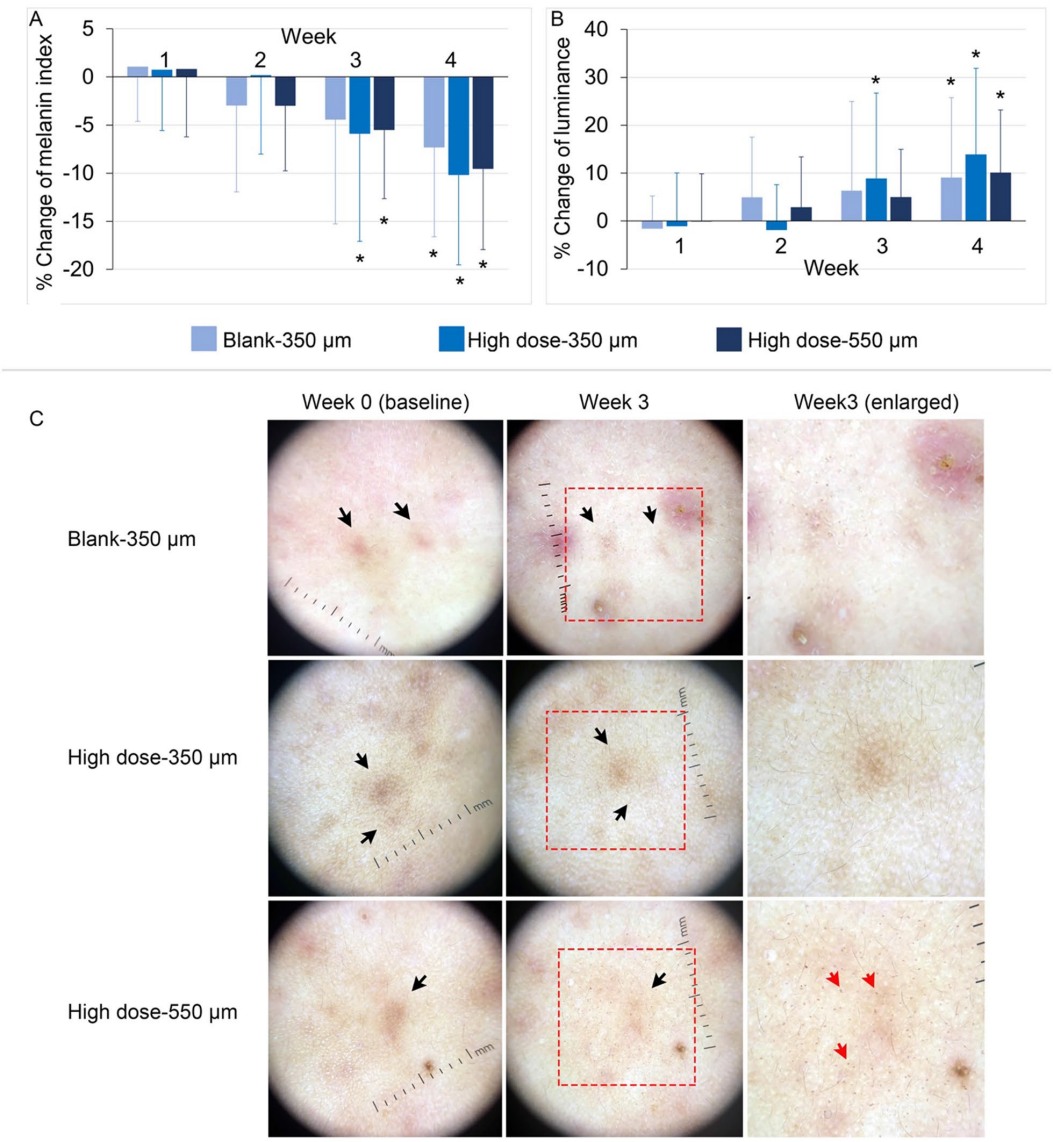
Skin whitening efficacy of vitC-gluta-DDMNs. Skin whitening of the post-acne-hyperpigmentation spots at 1, 2, 3 and 4 weeks after daily uses of the unloaded-DDMNs (Blank-350 µm), high dose-vitC-gluta-350-DDMNs (High dose-350 µm) and high dose-vitC-gluta-550-DDMNs (High dose-550 µm), is reported as % change of melanin index (**A**) and % change of luminance value L* (**B**) in relative to the original value (before treatment) of the same skin spot of each person. The bar represents the mean value, and the error bar represents standard deviation. Data were analyzed using mixed linear model by STATA software version 14.0 (Stata Corp LLC, USA). Significant difference between each time point and week 0 (baseline) is depicted with *at the *p* < 0.05. (**C**). Representative pictures of the post-acne-hyperpigmentation spots (black arrows) before the treatment (week 0, left column) and after 3 weeks of daily application of the tested sample (week 3, middle and right columns) are shown in (**C**) for the three tested samples which include unloaded-DDMNs (first row), high dose-vitC-gluta-350-DDMNs (second row) and high dose-vitC-gluta-550-DDMNs (third row). Red arrows point at examples of hyperpigmented spots formed after the microneedle treatment. The red dotted square in the middle column of C indicated the enlarged area shown in the right column.

We tested the three types of DDMNs, unloaded DDMNs with needle length of 350 µm, high dose-vitC-gluta-350-DDMN patch and high dose-vitC-gluta-550-DDMN patch, for their capability to erase post acne hyperpigmentation spot. Seventeen patients were enrolled, and eleven patients completed the study. Six patients could not complete the follow up due to COVID-19 pandemics situation in Bangkok. The mean age was 26.24 (SD 6.52) years old including of 5.88% of male and 94.12% of female. The skin type of the patients was 29.41% of skin type 3 and 70.59% of skin type 4 based on Fitzpatrick skin type system. The median of the onset post inflammatory hyperpigmentation was 3 months (IQR 1.5, 12). Here we report the result as % change of melanin index (Fig. 5A) and %change of luminance value (L*) (Fig. 5B) in relative to the original value of the same spot of each person at base line (week 0, the spot before treatment). Representative photographs of the treated spots are shown in Fig. 5C. Slight erythema after application was found on 2 patients who were treated with high dose-vitC-gluta-550-DDMN patch. However, the erythema spontaneously disappeared within 30 min after the application.

## Discussion

It is well accepted that vitC is an unstable compound^7^. To deliver vitC into epidermis and dermis by detachable dissolvable microneedles or DDMNs, we first made sure that vitC did not degrade in the DDMNs. The experiments were set up to observe the degradation of the vitC and to find the compound that could effectively stabilize vitC in the DDMNs (see Supplementary Information for details). It was found that vitamin E and coenzyme Q10 were incapable of stabilizing vitC whereas glutathione could excellently prevent vitC degradation. This result agrees well with the lower standard redox potentials of glutathione (− 0.24 V) than that of the vitC (+ 0.08 V)^37^. Co-loading of glutathione with vitC (at 25% glutathione relative to the amount of vitC) into the microneedles resulted in undetectable vitC degradation for at least 6 months when kept at 25, 40 or 50 °C which corresponds to a shelf-life of at least 28 months at room temperature as estimated based on Arrhenius equation (with assumption of constant activation energy)^38^.

We explored the mechanical properties of the fabricated DMNs (see Fig. 1B for the DMN images). Adding vitC and glutathione to the HA-PVA-sucrose needle did not significantly change the compressive strength of the three DMNs at the low compressive force rage of 0–20 N (Fig. 1C). Since the normal force used in finger pressing microneedles into skin is around 10–20 N^39–41^, this means that the unloaded, the low dose and the high dose vitC-gluta-DMNs should not break during normal use.

After that, the ex vivo skin penetration experiment was conducted. Because vitC is colorless, some red dyes were added to the DDMNs to aid the observation (Fig. 1B). The result clearly showed that all the fabricated DDMNs (low dose- and high dose-vitC-gluta-DDMNs of both needle lengths) could effectively penetrate porcine ear skin, and all the needles were embedded in the skin tissue (Fig. 1D).

To explore the diffusion of the cargo (delivered by DDMNs) in skin tissue, we performed ex vivo experiments in rat and pig skins. Comparing to a topical application of the solution containing the same amount of hydrophilic red dye, great improvement in intra-epidermal and intradermal delivery could be achieved via DDMNs (comparing the left and the right panels in Fig. 2A,B). When the dye solution was topically applied onto the rat skin, most dye molecules stayed on the stratum corneum during the first 30 min post the application. Little penetration of the red dye into skin tissue of rats via hair follicles was observe at 60- and 120-min post application. In contrast, immediately post DDMN administration to rat skin, an array of red dye spots could be clearly observed in the skin tissue. Cross sectional images of the skin tissue at 30 min post administration indicated obvious diffusion of the red dye from the originally observed spots at 0 min to the nearby area. In the rat skin, the no red dye horizontal gap of approximately 0.5 mm at 0 min was completely stained with red dye at 30 min. This implied that the horizontal diffusion of the red dye molecules in the rat skin was at least 0.25 mm/30 min (taking into accounts diffusion from the right and the left sides). Within 60 min post application, even distribution of the red dye in the epidermis and the upper dermis of the rat skin was observed (spanning approximately 0.4–0.5 mm depth from the stratum corneum) (Fig. 2A). Diffusion of dye from the upper dermis to the lower dermis, however, took place very slowly in our ex vivo rat skin experiment. At 120 min post administration, slight diffusion of the dye molecules into the lower dermis was observable but a majority of the dye molecules were still in the epidermis and the upper dermis. In contrast, diffusion of the hydrophilic red dye from upper dermis to the lower dermis took place more quickly in pig ear skin, i.e., even distribution of red dye in epidermis and the whole dermis (1 mm from stratum corneum) was observed at 120 min post application (Fig. 2B). The discrepancy between the rat and the pig skins was likely due to the different nature between the two skin tissues, i.e., lipid content in the lower dermis is higher in rat than in pig skin^42^. During the surgical cross section of both the pig and the rat skins at 30 min, we did not observe any rigid mass or gel-like mass of the embedded needles in the skin tissue. This likely implied that the needles completely dissolved in skin tissue at 30 min post the application. It should be noted here that no skin channel vestige from the microneedle penetration was observed in all cross-sectional skin tissue samples. This is likely due to the flexible and elastic nature of the fresh natural skin tissue material.

It should be noted here that in this work we used nail shaped needle with square column and square pyramidal tip. Previous work has revealed that triangular and square pyramidal shaped needles possess better penetration ability than the circular pyramidal shaped needles^39,40,43^. Therefore, here we used the square pyramidal tip. The nail shaped needles allow us to have increased needle volume without having to increase the size of the needle base. The increase in the needle volume means increased drug loading capacity. The sizes of our needle base were 180 × 180 and 200 × 200 μm squares for the 350 and the 550 μm needles, respectively. This small needle base contributes to the minimal skin penetration channel and easy resealing of the fresh and still elastic skin surface.

We explored the surface of human skin after the application of both the detachable DMNs (DDMNs) and the conventional DMNs (Fig. 4A, Supplementary Fig. S3). No abrasion was observed at the area applied with 350 μm DDMN, whereas the 550 μm DDMN produced doubtful negligible abrasion spots that disappeared on the next observation (30 min post application). However, application of the conventional DMNs (needles not detached from the base) of both needle lengths produced obvious surface abrasion spots at needle penetration sites. For the conventional DMNs, the longer the administration time, the more severity of the skin surface abrasion appeared. Skin surface disruption was most severed on skin underwent the longest period of having the DMN patch on (120 min). Our explanation on insignificant surface abrasion to no surface damage after the application of the detachable type of microneedles is that the quick detaching of needles from the base automatically pushed the embedded needles down from the skin surface, allowing the tissue at the surface to reseal (Fig. 4B left). With the opening at the skin surface of less than 2 min in the case of detachable microneedles and the small needle base (180 × 180 and 200 × 200 μm squares for the 350 and the 550 μm needles), elasticity and flexibility of the skin tissue material could prevent the development of any persisting skin surface hole. Unlike the detachable microneedles, administration of conventional DMNs involved pushing needle array into skin with the intact needles staying connected to their bases on skin surface and holding this position for a long time. This operation opened the skin surface at each needle penetration site for as long as the time each needle stayed undissolved and attached to the base. Such application prevented the resealing of skin surface, and consequently caused abrasion spot at the needle penetrating points (Fig. 4B right). It should be noted here that for the conventional DMNs, the needles did not completely dissolve in skin tissue within 2 h (longest time monitored). This is likely because there was not enough water in the skin tissue to completely dissolve the DMNs. It should be noted here that skin abrasion from the 30, 60 and 120 min application of the conventional DMN disappeared at approximately 30, 60, and 120–180 min after the removal of the patch, respectively (data not shown).

Both the unloaded DDMNs (blank-350-DDMNs) and the 350-DDMN patch containing 190 μg vitC and 47.5 μg glutathione (low dose-vitC-gluta-350-DDMNs) produced no sign of irritation when tested on the back skin of rats (Fig. 3). When the amounts of vitC and glutathione were increased to 380 μg vitC and 95 μg glutathione (high dose-vitC-gluta-350-DDMNs), doubtful negligible irritation (irritation index of grade 0.33 ± 0.82) was observed at day 1 post administration, but no sign of skin irritation was observed at day 7 post the single administration. Histopathological examination revealed no sign of inflammatory cell infiltration. When the length of the needles was increased to 550 μm, the unloaded DDMNs (blank-550-DDMNs) still produced no skin irritation response. However, the 550-DDMN patch containing 664 μg vitC and 166 μg glutathione (high dose-vitC-gluta-550-DDMNs) produced moderate irritation response (irritation index of grade 2 ± 1.27) at day 1 post the administration, with microscopically detectable infiltration of mononuclear cells (Fig. 3C). The irritation sign and the infiltration of mononuclear cells disappeared on the next examination which was day 7. Since the blank-550-DDMNs produced no sign of skin irritation, we have concluded that the irritation observed for the high dose-vitC-gluta-550-DDMNs was likely a result of high amount of vitC and glutathione delivered into the skin.

Since DDMNs are deposited into the dermis, safety of needle materials is most important. In this work, the water soluble HA and PVA polymers used as structural material for the DDMNs are non-crosslinked, therefore, can be soluble in tissue. Both polymers are biocompatible and biodegradable and have been approved by FDA in most countries for use inside human body. In vertebrates including Human, HA is naturally abundant in almost all biological fluids and tissues including dermis and epidermis. Up to the granular layer of the epidermis, hyaluronic acid forms a gel, facilitating the diffusion of nutrients, wastes and signalling molecules, which is of particular importance in tissue lacking blood and lymphatic vessels such as epidermis^44,45^. HA has a high turnover rate, mainly due to enzymatic hydrolysis of hyaluronidases enzyme^46^. HA has a halflife in Human tissue of 1.5 days^47^ and only 2–5 min in blood stream^48^. Biodegraded HA or fragmented HA is drained mostly into the lymphatic system, and some into the bloodstream^49,50^. The intravenous injection of ^14^C-HA into rat indicated that only a small portion of the HA was excreted in the urine, most HA was excreted within 24 h as CO_2_ via the respiration^51^. This indicates that the material is finally used as energy source. These information indicates that non-crosslinked HA is safe and can be degraded quickly if the material gets into the blood stream.

PVA is commonly used polymer in many medical devices and medicines due to its low protein adsorption characteristics, biocompatibility and high water solubility. Examples of common medical uses of PVA include soft contact lenses, eye drops, embolization particles, tissue adhesion barriers, artificial cartilages and oral tablets^52^. Injection of fluorescent dye-labeled PVA into rats showed that PVA in the blood circulation disappeared according to the first-order kinetics with half life of 7 h, and the material was eliminated via urinary and fecal excretion^53^.

The skin hypopigmentation efficacy of vitC-gluta-DDMNs was tested in vivo on the post acne-hyper-pigmented spots on the faces of 26.24 ± 6.52 year-old volunteers. Daily self-application of the tested sample at the studied spot was carried out by each volunteer. The studied spot was evaluated weekly via the measurement of melanin and L* using cutaneous colorimeter^54^.

The % change of the melanin index and the L* value are expressed relative to the values at the starting point (before treatment) of the same spot of each person. Decrease in the melanin index during the 4 week-tested period for all three samples, unloaded-DDMNs (Blank-350 µm), high dose-vitC-gluta-350-DDMNs (High dose-350 µm) and high dose-vitC-gluta-550-DDMNs (High dose-550 µm), although could be observed, statistically difference in the melanin reduction was observed at week 3 for only the high dose vitC-gluta-350-DDMNs and the high dose vitC-gluta-550-DDMNs groups. The control group showed statistically difference in melanin reduction at week 4 (Fig. 5A). The high dose vitC-gluta-350-DDMNs showed more pronounced melanin reduction than the high dose vitC-gluta-550-DDMNs group but not at statistically significant level. The faster reduction of melanin could be attributed to the vitamin C and glutathione delivered into the treated skin tissue. Likewise, increase in L* was observed for all three samples during the 4-week study period. Statistically significant increase in L* for the high dose vitC-gluta-350-DDMNs group started at week 3 whereas that of the high dose vitC-gluta-550-DDMNs group and the control group started at week 4. Among the three samples, high dose vitC-gluta-350-DDMNs group gave the fastest hypopigmentation effect. We speculate that the inferior of the high dose vitC-gluta-550-DDMNs group as compared to the high dose vitC-gluta-350-DDMNs group was likely a result of the micro-sized hyperpigmented spots generated by the deep needle penetration (red arrows in bottom right image of Fig. 5C). The pattern of the micro-sized hyperpigmentation spots matched the pattern of the needles. Such micro-sized hyperpigmentation spots were also observable in some cases for the control and the high dose vitC-gluta-350-DDMNs group, but the spots were very sporadic. We concluded that the 550 μm was too deep and could cause some skin micro damages that resulted in temporary hyperpigmentation. These results agreed with the slight temporary (30 min) erythema observed in two volunteers among the 17 volunteers who received the high dose vitC-gluta-550-DDMNs samples, but no erythema was observed in the other sample groups. Combining the result from the ex vivo experiment which clearly showed that drug diffusion could take place inside the skin tissue, the best hypopigmentation efficacy of the high dose vitC-gluta-350-DDMNs group, and the micro-hyperpigmentation spots observed in the high dose vitC-gluta-550-DDMNs group, we conclude that the hyperpigmentation can be treated by delivering vitC and glutathione into skin via detachable DMNs with the needle length of 350 μm.

It should be noted here that in this work we did not elucidate the actual distribution of the delivered vitC in the skin tissue. Since vitC has good water solubility, there is a possibility that vitC from the embedded microneedles might diffuse in the extracellular matrix of the skin tissue in the similar fashion to that of the water soluble red dye demonstrated in the ex vivo experiment above (Fig. 2). The uptake of the delivered vitC (diffused from the embedded microneedles) by local skin cells such as melanocytes, fibroblasts and keratinocytes should take place easily since the normal supply of nutrients to these cells usually relies on the diffusion from the vasculars in the dermis through extracellular matrix^55^.

With an average thicknesses of facial epidermis and dermis of ~ 40–50 and ~ 1000 μm, respectively^56^, the daily administration of vitC-gluta-DDMNs of 350 µm likely had the vitC delivered into epidermis and upper dermis of the facial skin (assuming similar skin tissue between Human’s and pig’s, see the ex vivo result in Fig. 3), and it was indeed the targeted location for vitC to induce hypopigmentation (skin whitening). Previous studies have reported that not only the epidermal‐melanocyte-keratinocyt orchestration unit but also the fibroblasts in the dermal layer play an active role in regulating skin pigmentation^57–60^. Since the delivered vitC likely spanned from stratum corneum to the depth of 400 μm for the 350-DDMNs, the volume of the skin tissue (administered with the 1.76 cm^2^ patch) saturated with the delivered vitC would be 0.704 cm^3^. Assuming an average density of epidermis-dermis tissue of around 0.95 g/cm^3^, an estimated amount of vitC delivered into the epidermis-upper dermis is 270 μg vitC/g of tissue. Previous works have reported vitC concentration ranges in human skin of 60–640 μg/g of fresh epidermis and 30–130 μg/g of fresh dermis^1,61,62^. From the above estimation, vitC delivered into epidermis should still be toloratable, thus explaining the no skin irritation observed for the high dose vitC-gluta-350-DDMNs group. Although, the amount of vitC delivered into the upper dermis might exceed the upper range naturally found in dermis, no irritation was observed. It is possible that the dermis can tolorate higher concentration of vitC than the normally found value.

The post-acne-hyperpigmentation is the result of skin tissue injuries from acne and likely is regulated by infection, inflammation and immune cells^63,64^. Here the DDMN treatment was carried out at the post acne period in which all the inflammation had already been cleared. Supplying vitC to the post-injured spot could help stimulating tissue regeneration and simultaneously blocking the melanogenesis, all of which led to fast generation of new tissue with less accumulation of melanin. This led to a lighter color skin appearance at the vitC-gluta-DDMNs treatment spots as compared to the control spots.

In conclusion, in this work we have demonstrated that with glutathione, vitamin C can be stabilized in the microneedles. Combining the detachable dissolveable microneedle platform with stable vitC-glutathione formulation and microneedles of good skin penetration ability, effective delivery of vitamin C and glutathione into epidermis and dermis to lessen local skin hyperpigmentation in Human could be achieved. With short application time of 2 min, resealing of skin surface takes place quickly and surface skin damage can be avoided.

## Methods

### Fabrication of dissolvable microneedles or DMNs for mechanical testing

Dissolvable microneedles or DMNs were fabricated at room temperature under laminar flow, in a clean room and associated controlled environments (Biocontamination control, ISO 14698-1:2003 en), using the procedure as previously described^34^. The polymer solution was autoclaved at 121 °C for 15 min before use. Briefly, appropriate volume of sterile polymer solution (2.08% HA of MW 5000 Da, 4.16% PVA of MW 32,000, 4.16% sucrose, in weight to volume of water) was poured into silicone mold and the filled mold was left under the moisture control atmosphere of 5% humidity until dry. VitC-gluta-DMNs were prepared similarly except that vitC and glutathione were added to the polymer solution. DMNs of two different needle lengths were fabricated, the 350 µm DMN patch (1.5 cm in diameter or 1.76 cm^2^ circular patch containing 89 needles arranged with the tip to tip distance of 1120 μm; each needle is nail-shaped with 180 × 180 × 200 μm (W × L × H) square column and 150 μm height of the square pyramid on the top), and the 550 µm DMN patch (1.5 cm in diameter or 1.76 cm^2^ circular patch containing 89 needles arranged with the tip to tip distance of 1110 μm; each needle is nail-shaped with 200 × 200 × 400 μm (W × L × H) square column and 150 μm height of the square pyramid on the top). The needles were sitting on a 500 µm thick base made of the same material as the needles. All prepared DMNs were examined under stereomicroscope (Olympus DP22, Tokyo, Japan). Concentrations of vitC and glutathione, dimension of the DMN and their code name used in this paper are shown in Table 1.

**Table 1:** The characteristic and active ingredients of tested microneedle.

Prefix of the names of the DMNs or DDMNs	Needle length (μm)	The percentage of active ingredients in needle matrix	The amount of active ingredients/patch
High dose-vitC-gluta-350-	350	40% vitC 10% glutathione	380 μg vitC 95 μg glutathione
High dose-vitC-gluta-550-	550	40% vitC 10% glutathione	664 μg vitC 166 μg glutathione
Low dose-vitC-gluta-350-	350	20% vitC 5% glutathione	190 μg vitC 47.5 μg glutathione
Low dose-vitC-gluta-550-	550	20% vitC 5% glutathione	332 μg vitC 83 μg glutathione

### Fabrication of detachable dissolvable microneedles or DDMNs for stability, ex vivo, in vivo and clinical test experiments

Detachable DMNs (so called DDMNs) were fabricated in the cleanroom under laminar flow using the micro-molding method in a similar fashion to the above DMN preparation except that the base of needles was attached to Whatman No. 1 filter paper (Cytiva, USA). Briefly, for red dye-loaded DDMNs, appropriate volume of sterile polymer solution containing red dye (2.08% HA of MW 5000 Da, 4.16% PVA of MW 32,000, 4.16% sucrose, 2.52% ponceau 4R, 1.56% azorubine in weight to volume of water) were poured into silicone mold and the filled mold was left under the moisture control atmosphere of 5% humidity until stable gel was formed. Then the Whatman filter paper was attached and the DDMN patch were put under moisture control atmosphere of 2.5% humidity until completely dry. VitC-gluta-DDMNs were prepared similarly except that the dye solution was replaced with solution containing vitC and glutathione. Two different lengths of needle of DDMN patches were fabricated, the 350 µm DDMN patch (1.5 cm in diameter or 1.76 cm^2^ circular patch containing 89 needles arranged with the tip to tip distance of 1120 μm; each needle is nail-shaped with 180 × 180 × 200 μm (W × L × H) square column and 150 μm height of the square pyramid on the top), and the 550 µm DDMN patch (1.5 cm in diameter or 1.76 cm^2^ circular patch containing 89 needles arranged with the tip to tip distance of 1110 μm; each needle is nail-shaped with 200 × 200 × 400 μm (W × L × H) square column and 150 μm height of the square pyramid on the top). All prepared DDMNs were examined under stereomicroscope. Samples from each DDMN batches were tested for microbial contamination (aerobic mesophilic bacteria, yeast and mould, *Pseudomonas aeruginosa*, *Staphylococcus aureus* and *Candida albicans*) at the standard microbiological laboratory, Department of Microbiology, Biodernat Co., Ltd., Thailand. Concentrations of vitC and glutathione, dimension of the DDMN and their code name used in this paper are shown in Table 1.

### Mechanical property of DMNs

Compressive strength of DMN arrays was determined by measuring displaced distance as the tested sample was compressed at an applied axial force rate of 1 mm/min (with the maximum force of 100 N or until microneedle broke) through a universal testing machine (Shimadzu EZ-S, Shimadzu Corporation, Tokyo, Japan) operated at 32 °C and a relative humidity of 7.4 ± 2%. The tested DDMN samples are shown in Table 1.

### Ex vivo skin penetration and diffusion

Full thickness of fresh porcine (crossbred pig, *Sus scrofa domesticus*) ear skin and fresh Wistar rats (*Rattus norvegicus*) dorsal skin (obtained from Department of Pathology, Faculty of Veterinary Science, Chulalongkorn University, and National Laboratory Animal Center, Mahidol University, Thailand, respectively) were cleaned with water, shaved with hair clipper, and dried with tissue paper. All fresh skin pieces were used immediately. All ex vivo skin experiments were started within 2 h post the animal death.

Skin penetration of the low dose-vitC-gluta-350-DDMN patch, and the low dose-vitC-gluta-550-DDMN patch was investigated. Red dye, vitC and glutathione were co-loaded into the DDMNs. The amounts of dyes were 9.97 and 6.17 µg of ponceau and azorubine in each of the low dose-vitC-gluta-350-DDMN, and 17.43 and 10.79 µg of ponceau and azorubine in each of the low dose-vitC-gluta-550-DDMN patch. The experiment was initiated by pressing the DDMN patch against the stratum corneum side of the porcine skin piece with the force of 20 N. This pressing of the DDMN patch against the skin took approximately 10 s. Then the backing of the DDMNs was wet with 2 drops of water and the patch was hand-pressed against the skin for another 2 min before removing the backing from the skin. The skin was immediately cross sectioned along the row of embedded red dye spots, and the sectioned skin was straightaway visualized under the stereomicroscope.

Diffusion test was performed using red dye-loaded DDMNs. The red dye-loaded DDMN patch used in the experiment was the 550 µm needle-height-DDMNs containing 470 µg dye/patch (290 µg ponceau and 180 µg azorubine). The DDMNs were applied to the skin as described above, then the skin pieces were placed on wet filter paper (soaked with 0.01 M PBS buffer of pH 7.4) at room temperature (32 °C). At designated times (0, 30, 60, 120 min post administration), the skin piece was surgically sectioned along the row of the embedded needle spots, and the sectioned skin piece was immediately visualized under the stereomicroscope. Each control skin piece was topically applied with the red dye solution containing the same amount of dye as in each DDMN patch (470 µg dye in 20 µL water spread on 1.76 cm^2^ area of the skin) and the skin was processed similarly.

### Observation of skin surfaces of human volunteers

The in vivo skin surface monitoring experiment on human volunteers was carried out using the low dose-vitC-gluta-350-DDMN patch and the low dose-vitC-gluta-550-DDMN patches. DDMN application was carried out at the inner forearm skin of four volunteers. Each volunteer was administered with both DDMN samples (2 min application time) at location 2 cm apart. The skin sites were photographed by stereomicroscope at various times (0, 30, 60, 120 min) post the administration. Control skin (the nearby area with no DDMN administration) was also photographed.

The same volunteer was also administered with four conventional DMNs at location 2 cm apart. The DMN administration was carried out with the DMN patch being left on the skin (no needle detachment feature). Each DMN patch was removed at different times, 0, 30 60 and 120 min post the initial application. Skin surface photograph was taken immediately after the removal of the DMN patch.

### Optimization of DDMN formulation to stabilize the loaded vitC

#### Stability of vitC in solution

Three antioxidants including coenzyme Q10, vitamin E and glutathione, were experimented for their effect on vitC stability^65–67^. VitC solutions containing various amounts of each antioxidant were prepared. Final concentration of vitC was 0.002% (w/v) in water and final concentrations of the tested antioxidant were 0.002, 0.001, 0.0005, 0.00025 and 0.000125% (w/v). The tested solution was kept in normal glass test tube under normal natural indoor light (UVB (280–320 nm) of 0.05–0.06 mW/cm^2^ and UVA (320–400 nm) of 3.0–5.0 mW/cm^2^), and at designated time the solution was subjected to UV–Vis absorption analysis (maximum absorption of vitC is 265 nm) using the CARY 100 Bio UV–Vis spectrometer (Palo Alto, USA). Another set of the same sample was kept in the dark and analyzed similarly.

#### VitC stability in DDMNs

Each of the freshly prepared DDMNs co-loaded with glutathione at 25% of the amount of vitC (low and high dose-vitC-gluta-350-DDMN patch) was kept separately in individually sealed foil package at 25 (RT), 40 and 50 °C. At specific time points, the DDMN patches were taken out and dissolved in 10 mL water under light-proof condition. The obtained solutions were immediately subjected to UV–Vis absorption analysis as described above. At each time point, freshly prepared vitC standard solutions were used to construct calibration curve. Experiment was carried out in triplicate.

### In vivo skin irritancy

All animal experiments were approved by Chulalongkorn University Institutional Animal Care and Use committee (CU-IACUC) (Protocol no. 1973015). The study was carried out in compliance with the ARRIVE guidelines 2.0 checklist (Table S1) and all methods were carried out in accordance with the guidelines and CU-IACUC regulations. The small sample size was selected to minimize the numbers of animal used. Eighteen male Wistar rats (*Rattus norvegicus*) with the weight of 300–400 g) (Nomura Siam International Co., Ltd., Bangkok, Thailand) were housed in groups of two and given 2 weeks to acclimate to the housing environment (temperature: 22 ± 2 °C, humidity: 50 ± 20% and a standard 12:12 light: dark cycle). Eighteen fourteen-week-old-male-Wistar rats were randomly divided into 2 groups: Groups of day 1 and day 7. Five types of DDMN samples were tested for skin irritation using in vivo skin at the back of rats. These groups were (1) low dose-vitC-gluta-350-DDMN patch, (2) high dose-vitC-gluta-350-DDMN patch), (3) high dose-vitC-gluta-550-DDMN patch, and (4 and 5) unloaded DDMNs of 350 and 550 μm needle lengths.

Prior to DDMN administration, rats were anesthetized. The hairs on the dorsal back skin were trimmed with hair clipper (less than 1 mm length) and the shaved skin was cleaned and dry. DDMNs were administered (as described above) randomly regarding the location at the back of the rats by a veterinarian using double blind technique. Gross and histopathological aspects were observed. Each animal was randomly administered with the maximum of 3 DDMN patches on its back.

For gross pathology, the gross lesions were assessed over the signs of skin reaction on day 1 and day 7 post single administration using the procedure of ISO 10993-10: Biological evaluation of medical devices-Part 10, Tests for irritation and skin sensitization (Draize test) and OECD 404 guideline for testing of chemicals (acute dermal irritation and corrosion). The irritation score concerning the signs of skin erythema and edema after DDMN application was calculated by dividing the sum of irritation scores of all animals in each group with the total number of animals in that group. All irritation scores, the cumulative irritation indexes, were categorized according to the following criteria: 0–0.4: negligible irritation, 0.5–1.9: slight irritation, 2–4.9: moderate irritation, 5–8: severe irritation, by 2 independent veterinary pathologists. The scoring system used for skin reaction is shown in Table S2 in the Supplementary Information or SI.

For histopathology, the full thickness of skins from all experimental groups on day 1 and day 7 post single administration were collected and fixed in 10% buffered formalin before processed by the routine hematoxylin & eosin (H&E) staining and observed under the light microscope by 2 independent veterinary pathologists using single blind technique. The skin samples were evaluated in terms of epidermal and dermal parameters that modified from Machtinger et al.^68^ and ISO 10993-10:2010.

### Treatment of hyperpigmentation in human

Hypopigmentation efficacy of DDMN patches was evaluated in human volunteers through a randomized triple blind-controlled study, conducted at Srinakharinwirot skin center, Bangkok, Thailand during July–August 2021. The study was approved by the research ethic committee of Srinakharinwirot University (SWEC/F-095/2564) and was later registered in the Thai Clinical Trial Registry (TCTR20210903004) on 03/09/2021. All methods were performed in accordance with the relevant guidelines and regulations of the Declarations of Helsinki. Patients who have had allergic reaction to vitC or glutathione, history of keloid, concurrent skin infection on face, previous used of any medication or laser that have whitening effect (hydroquinone, arbutin, azelaic acid, kojic acid, licorice extract, Lakoocha extract, vitC, vitamin E, soybean extract, resorcinol, alpha/beta-hydroxy acid, nicotinamide) for 1 week before enrolled, were excluded. Seventeen patients were enrolled, and eleven patients completed the study. Six patients could not complete the follow up due to COVID-19 pandemics situation in Bangkok, Thailand. All volunteers were over 18 years old who had more than 3 lesions of post inflammatory hyperpigmentation from acne on their face. Informed written consent was obtained from each volunteer before the study.

The post inflammatory hyperpigmented lesions on the face of each volunteer were divided into three groups, lesion A, B and C, by a computer-generated random sequence: lesion A was treated by unloaded-350-DDMNs, lesion B was treated by high dose-vitC-gluta-350-DDMN patch, and lesion C was treated by high dose-vitC-gluta-550-DDMN patch.

Volunteers applied the appropriated DDMNs on the designated clean and dry studied area by themselves. DDMN patch was applied daily before bedtime for 4 weeks. During the entire study period, volunteers had normal routine of skin cleansing and makeup. Personal and medical history was obtained at baseline. Colorimeter (DSMIII ColorMeter, Cortex technology, Denmark) was used to assess melanin index and luminance (L*) at week 0 (baseline), 1, 2, 3 and 4. Melanin index represents skin darkness with scale of 0 (white) to 100 (total black). The L* represents skin lightness with scale of 0 (total black) to 100 (white). The % change of melanin index and % change of L* were calculated as follows:

$$\% {\text{ Change of melanin index }} = \, \left[ {\left( {{\text{melanin index Wt }}{-}{\text{ melanin index W}}0} \right)/{\text{melanin index W}}0} \right)] \times {1}00,$$

$$\% {\text{ Change of L}}^{*} \, = \, \left[ {\left( {{\text{L}}^{*} {\text{ Wt }}{-}{\text{ L}}^{*} {\text{ W}}0} \right)/{\text{ L}}^{*} {\text{ W}}0} \right)] \times {1}00,$$

where W0 = week 0, Wt = week of the measurement.

For skin lightening or hypopigmentation, melanin index should decrease and L* should increase.

### Statistical analysis

The data from animal study were analyzed by one-way ANOVA followed by the Tukey’s multiple comparison post hoc test using GraphPad Prism (version 5.03) software (GraphPad, USA). The data from human clinical study were analyzed by mixed linear model using STATA (version 14.0) software (Stata Corp LLC, USA). Differences were considered significant at p < 0.05.

## Supplementary Information

Supplementary Information.
