# Supplementary material for: Detachable dissolvable microneedles: intra-epidermal and intradermal diffusion, effect on skin surface, and application in hyperpigmentation treatment
Source: Sci Rep. 2021 Dec 16;11:24114. doi: 10.1038/s41598-021-03503-5 (PMC8677736; doi:10.1038/s41598-021-03503-5)
Supplement: Supplementary file 1 — Supplementary Information. [file 41598_2021_3503_MOESM1_ESM.docx]

Supplementary information for

**Detachable dissolvable microneedles: Intra-epidermal and intradermal diffusion, Effect on skin surface, and Application in hyperpigmentation treatment**

Pritsana Sawutdeechaikul^1^, Silada Kanokrungsee^2^, Thanyapat Sahaspot^2^, Kamonwan Thadvibun^2^, Wijit Banlunara^3,7^, Benchaphorn Limcharoen^4,7^, Titiporn Sansureerungsikul^5^, Teeranut Rutwaree^5^, Miranda Oungeun^6^, Supason Wanichwecharungruang^1,7^*

^1^Department of Chemistry, Faculty of Science, Chulalongkorn University, Thailand.

^2^Skin Center, Faculty of medicine, Srinakharinwirot University, Thailand

^3^Department of Pathology, Faculty of Veterinary Science, Chulalongkorn University, Thailand.

^4^Department of Anatomy, Faculty of Veterinary Science, Chulalongkorn University, Thailand

^5^Mineed Technology, 142 Innovation Cluster 2, Thailand Science Park, Pathum Thani, Thailand.

^6^Department of Petrochemistry and Polymer Science, Faculty of Science, Chulalongkorn University, Thailand

^7^Center of Excellence in Materials and Bio-interfaces, Chulalongkorn University, Thailand.

*Corresponding author: Supason Wanichwecharungruang, supason.p@chula.ac.th

**Stability of Vitamin C in solution and in DDMN patch**

*Results*

Amounts of vitC remained in aqueous solutions (starting concentration of 0.002% w/v) kept at room temperature (~25 ºC) under normal indoor daylight (UVB (280–320 nm) of 0.05–0.06 mW/cm^2^ and UVA (320–400 nm) of 3.0–5.0 mW/cm^2^) are shown in Supplementary Fig. S1 A. Effects of coenzyme Q10, vitamin E and glutathione (at concentrations of 0.002, 0.001, 0.0005, 0.00025 and 0.000125% (w/v) which correspond to 100, 50, 25, 12.5 and 6.25% of the starting amount of vitC in the solution) on the degree of vitC degradation, are shown in the same graph. The degradation rate of vitC in aqueous solutions (starting vitC concentration of 0.002% w/v) containing glutathione (at 25% of the amount of starting vitC) and kept at various temperatures under light-proof condition, are shown in Supplementary Fig. S1 B. When the same 0.002% w/v vitC solution was kept in the dark for 4 h, the amount of vitC remained the same during the 4 week monitoring period (data not shown).

When vitC was incorporated into the solid matrix of DDMNs and kept at 40 ºC, a presence of glutathione in the matrix at 25% of the amount of starting vitC could prevent the degradation of vitC for up to 12 weeks (the longest period tested in the experiment), whereas approximately 30% of vitC degradation was observed at week 12 in the sample with no glutathione (Supplementary Fig. S1 C). Stability profiles of vitC in DDMNs co-loaded with glutathione (at 25% of the amount of vitC) kept at various temperatures for 18 weeks are shown in Supplementary Fig. S1 D.


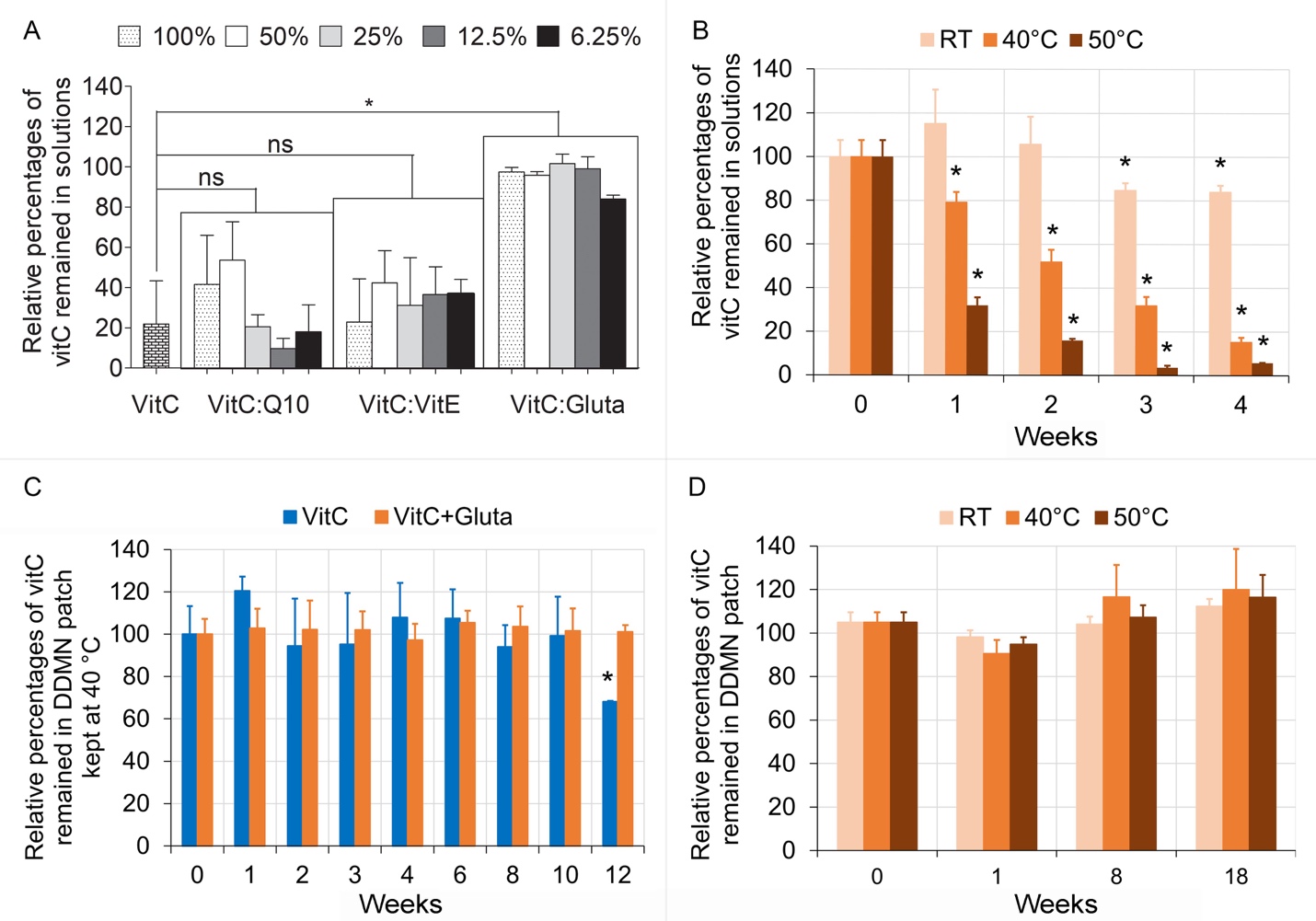


Figure S1. Effect of various antioxidants on vitC stability in aqueous solution (A and B) and solid DDMNs (C and D). **Graph A** shows relative percentages of vitC remained in the solutions after being kept at room temperature (~25 ºC) under normal daylight for 4 h (UVB, 280–320 nm, of 0.05–0.06 mW/cm^2^ and UVA, 320–400 nm, of 3.0–5.0 mW/cm^2^) for 0.002% (w/v) pure vitC solution (vitC), and 0.002% (w/v) vitC solutions containing coenzyme Q10 (vitC:Q10), vitamin E (vitC:vitE) and glutathione (vitC:Gluta) at the concentrations of 0.002, 0.001, 0.0005, 0.00025 and 0.000125% (w/v) (correspond to 100, 50, 25, 12.5 and 6.25% of the starting amount of vitC in the solution). **Graph B** shows relative percentages of vitC remained in the vitC solutions with starting vitC concentration of 0.002% (w/v) and glutathione at 25% of the amount of the starting vitC, after being kept under light-proof condition for various weeks, at room temperature, 40 and 50 ºC. **Graph C** shows amount of vitC remained in the DDMN patches containing 20% vitC and 5% glutathione in the needle matrix (corresponds to glutathione at 25% of the amount of vitC, labeled in the graph as VitC+Gluta) and in control DDMN patch containing only vitC (containing 20% vitC in the needle matrix, labeled in the graph as VitC), after being kept under light-proof condition at 40 °C for various times. **Graph D** shows relative percentages of vitC remained in the DDMNs (containing 40% vitC and 10% glutathione in the needle matrix which corresponds to glutathione at 25% of the amount of vitC) kept under light-proof condition at room temperature, 40 and 50 °C for various times. Data are shown as mean ± SD, obtained from three independent experiments. Statistical analysis at the significant level of p<0.05 was carried out by one-way ANOVA followed by Tukey post hoc test for graph A (* represents significant difference whereas n.s. represents insignificant difference), and by two-way ANOVA followed by Bonferroni post hoc test for graphs B, C and D (* on each bar indicates significant difference between that time point and time 0, the bars with no * are insignificant different from time 0).

*Discussion*

We observed fast degradation of vitC when kept as 0.002% (w/v) aqueous solution at room temperature under normal indoor daylight, i.e., around 80% of the compound degraded after 4 h period (Supplementary Fig. S1 A in SI). Under such condition, both vitamin E and coenzyme Q10 were incapable of stabilizing vitC even when used at the same amount of vitC. In contrast, glutathione showed excellent ability to stabilize vitC even when used at only 6.25% of the amount of vitC. The standard redox potentials (from oxidized form to reduced form) of vitamin E, coenzyme Q10, vitamin C and glutathione are +0.37, +0.10, +0.08 and -0.24 volts, respectively^1^. Among the three antioxidants tested, only glutathione possesses lower standard redox potential comparing to that of vitC. Therefore, unsurprisingly, it is the only compound that could prevent the oxidation of vitC. Our experiment showed that when added with glutathione at 25% of the amount of vitC, increased stability of vitC was observed for solution kept at room temperature but not for solutions kept at 40 and 50 ºC (Supplementary Fig. S1 B). VitC kept in solid matrix of the DDMN patch showed slower degradation than that kept in solution (comparing Supplementary Fig. S1 B and C). This is because the chemical reaction is less probable in solid than in liquid state. Co-loading of glutathione with vitC into DDMN matrix could prevent vitC degradation even when the DDMN patches were kept at 50 ºC for 18 weeks (the longest observation period in this experiment). Via Arrhenius equation on temperature dependent reaction rate of chemical^2^, the observed 18 week-stability of vitC (containing 25% glutathione in relative to the amount of vitC) in the DDMN patch at the accelerated condition of 50 ºC could be approximated to 28 months at room temperature. Such high vitC stability was achieved through the combined effect of slower degradation under solid condition and strong reducing power of glutathione.

­­­

References

1 Vasdev, S., Gill, V. & Singal, P. Modulation of oxidative stress-induced changes in hypertension and atherosclerosis by antioxidants. *Exp. Clin. Cardiol.* **11**, 206-216 (2006).

2 *ASTM F1980-16, Standard Guide for Accelerated Aging of Sterile Barrier Systems for Medical Devices*. (ASTM international 2016).


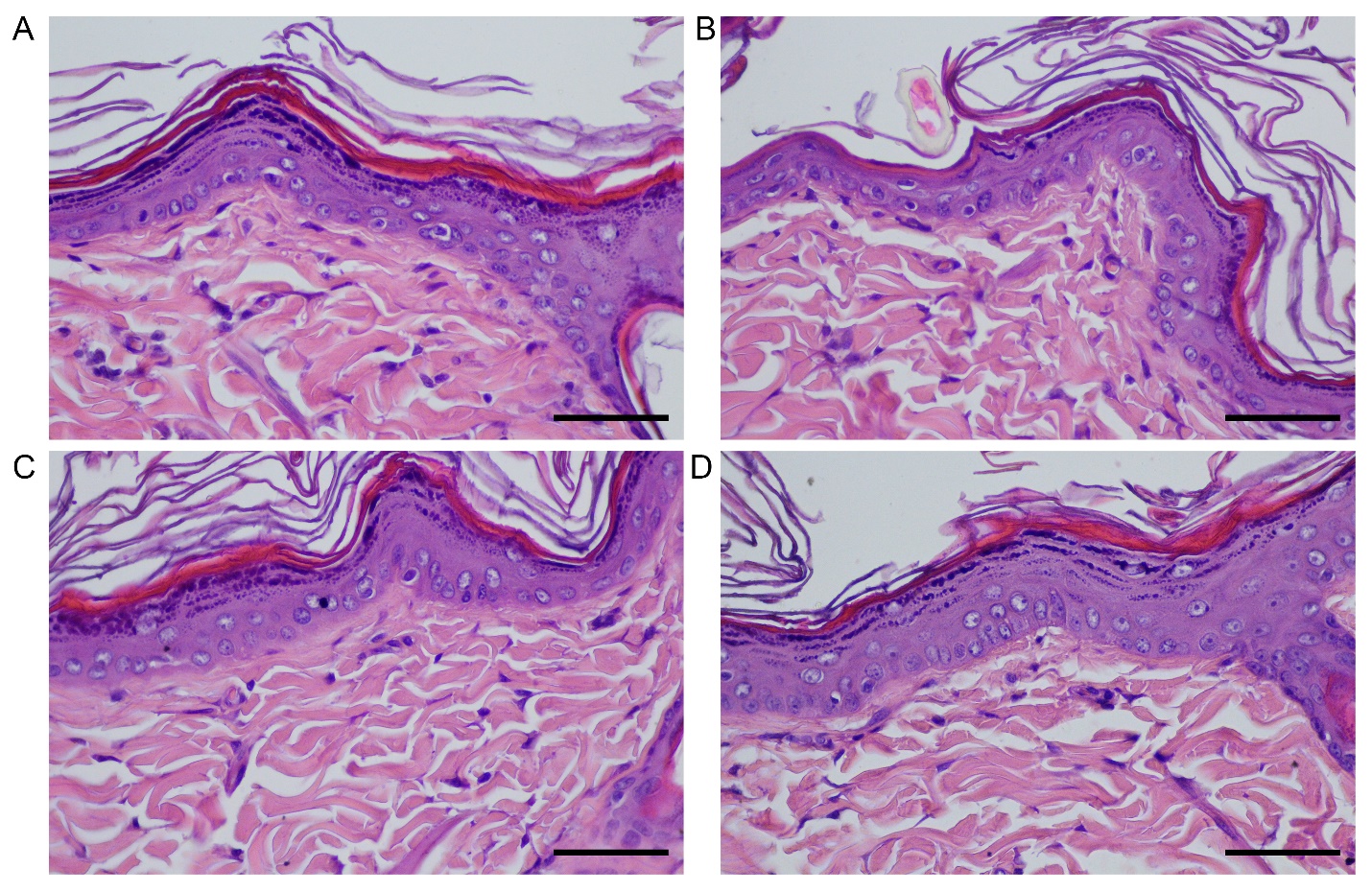


Figure S2. Representative pictures of the histopathology of rat skin at day 7 after the single DDMN administration. A) Unloaded-550-DDMNs B) Low dose-vitC-gluta-350-DDMNs C) High dose-vitC-gluta-350-DDMNs D) High dose-vitC-gluta-550-DDMNs. Scale bar represents 50 µm.


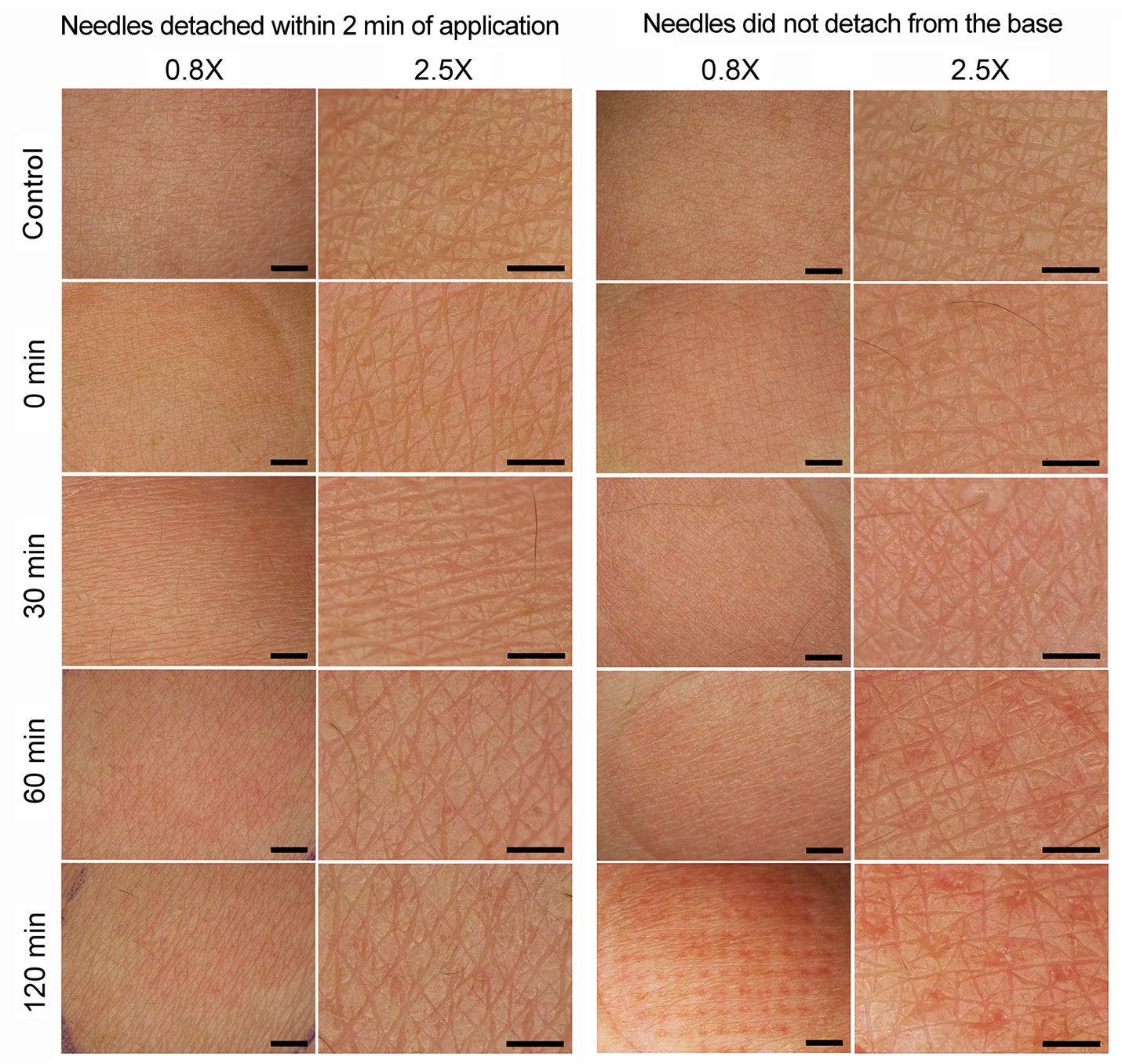


Figure S3. Photographs at 0.8X (first and third columns from the left) and 2.5X (second and fourth columns from the left) magnifications of a human volunteer inner forearm skin after the application of microneedles with 550 μm needle height. ***For images on the first and the second columns from the left***, the skin was applied with the low dose-vitC-gluta-550-DDMNs using detaching mechanism (the needles were detached from the base within 2 min of application) and the skin was photographed at various times post the administration (see the denoted times on the left of each row). ***For images on the third and the fourth columns from the left,*** the skin were applied with the low dose-vitC-gluta-550-DMNs with no detaching mechanism (the DMN patch was pressed on the skin pieces for 0, 30, 60 and 120 min) and then the skin was photographed (see the denoted times on the left of each row). Control is the image of the skin that was not administered with DMNs. Scale bar on each image of the first and the third columns from the left represents 2 mm. Scale bar on each image of the second and the fourth columns from the left represents 1 mm.

Table S1. The ARRIVE guidelines 2.0: author checklist

| The ARRIVE Essential 10 | | | |
| --- | --- | --- | --- |
| Item |  | Recommendation | Section, or reason for not reporting |
| Study design | 1 | For each experiment, provide brief details of study design including:   1. The groups being compared, including control groups. If no control group has been used, the rationale should be stated. | Methods, first paragraph under *in vivo* skin irritancy section |
|  |  | 1. The experimental unit (e.g. a single animal, litter, or cage of animals). | Methods, first paragraph under *in vivo* skin irritancy section |
| Sample size | 2 | 1. Specify the exact number of experimental units allocated to each group, and the total number in each experiment. Also indicate the total number of animals used. | Methods, first paragraph under *in vivo* skin irritancy section |
|  |  | 1. Explain how the sample size was decided. Provide details of any a priori sample size calculation, if done. | Methods, first paragraph under *in vivo* skin irritancy section |
| Inclusion and exclusion criteria | 3 | 1. Describe any criteria used for including and excluding animals (or experimental units) during the experiment, and data points during the analysis. Specify if these criteria were established a priori. If no criteria were set, state this explicitly. | All collected data were used in the analysis with no criteria. |
|  |  | 1. For each experimental group, report any animals, experimental units or data points not included in the analysis and explain why. If there were no exclusions, state so. | No exclusion |
|  |  | 1. For each analysis, report the exact value of n in each experimental group. | Fig.3 |
| Randomisation | 4 | 1. State whether randomisation was used to allocate experimental units to control and treatment groups. If done, provide the method used to generate the randomisation sequence. | Methods, first and second paragraphs under *in vivo* skin irritancy section |
|  |  | 1. Describe the strategy used to minimise potential confounders such as the order of treatments and measurements, or animal/cage location. If confounders were not controlled, state this explicitly. | Methods, first and second paragraphs under *in vivo* skin irritancy section |
| Blinding | 5 | Describe who was aware of the group allocation at the different stages of the experiment (during the allocation, the conduct of the experiment, the outcome assessment, and the data analysis). | Methods, fourth paragraph in the *in vivo* skin irritancy section |
| Outcome measures | 6 | 1. Clearly define all outcome measures assessed (e.g. cell death, molecular markers, or behavioural changes). | Methods, second, third and fourth paragraphs in the *in vivo* skin irritancy section |
|  |  | 1. For hypothesis-testing studies, specify the primary outcome measure, i.e. the outcome measure that was used to determine the sample size. | Small sample size was used to minimize the numbers of animal used. |
| Statistical methods | 7 | 1. Provide details of the statistical methods used for each analysis, including software used. | Method, statistical analysis section and Fig. 3 |
|  |  | 1. Describe any methods used to assess whether the data met the assumptions of the statistical approach, and what was done if the assumptions were not met. | Methods, third and fourth paragraph in the *in vivo* skin irritancy section and Fig. 3 |
| Experimental animals | 8 | 1. Provide species-appropriate details of the animals used, including species, strain and substrain, sex, age or developmental stage, and, if relevant, weight. | Methods, first paragraph under *in vivo* skin irritancy section |
|  |  | 1. Provide further relevant information on the provenance of animals, health/immune status, genetic modification status, genotype, and any previous procedures. | Methods, first paragraph under *in vivo* skin irritancy section |
| Experimental procedures | 9 | For each experimental group, including controls, describe the procedures in enough detail to allow others to replicate them, including:   1. What was done, how it was done and what was used. | Methods, first and second paragraphs under *in vivo* skin irritancy section |
|  |  | 1. When and how often. | Methods, third and fourth paragraphs in the *in vivo* skin irritancy section |
|  |  | 1. Where (including detail of any acclimatisation periods). | Methods, first paragraph under *in vivo* skin irritancy section |
|  |  | 1. Why (provide rationale for procedures). | Methods, *in vivo* skin irritancy section |
| Results | 10 | For each experiment conducted, including independent replications, report:   1. Summary/descriptive statistics for each experimental group, with a measure of variability where applicable (e.g. mean and SD, or median and range). | Fig. 3 |
|  |  | 1. If applicable, the effect size with a confidence interval. | - |

Table S2. Criteria for gross pathological evaluations

Scoring system for skin reaction (ISO 10993-10:2010)

| Reaction | Irritation score |  |
| --- | --- | --- |
| Erythema and eschar formation | | |
| No erythema | 0 |  |
| Very slight erythema (barely perceptible) | 1 |  |
| Well-defined erythema | 2 |  |
| Moderate erythema | 3 |  |
| Severe erythema (beet-redness) to eschar formation | 4 |  |
| Edema formation | | |
| No edema | 0 |  |
| Very slight edema (barely perceptible) | 1 |  |
| Well-defined edema (edge of area well-defined by finite raising) | 2 |  |
| Moderate edema (raised approximately 1 mm.) | 3 |  |
| Severe edema (raised more than 1 mm. and extending beyond exposure area) | 4 |  |
| Maximal possible score for irritation | 8 |  |
| Other adverse changes at the skin sites shall be recorded and reported |  |  |
